# A Near-Infrared Light Triggered Composite Nanoplatform for Synergetic Therapy and Multimodal Tumor Imaging

**DOI:** 10.3389/fchem.2021.695511

**Published:** 2021-07-22

**Authors:** Mingzhou Wu, Shuqing He, Xin Hu, Jingqin Chen, Enna Ha, Fujin Ai, Tao Ji, Junqing Hu, Shuangchen Ruan

**Affiliations:** ^1^ Shenzhen Key Laboratory of Laser Engineering, College of Physics and Optoelectronic Engineering, Shenzhen University, Shenzhen, China; ^2^ College of Health Science and Environmental Engineering, Shenzhen Technology University, Shenzhen, China; ^3^ Sino-German College of Intelligent Manufacturing, Shenzhen Technology University, Shenzhen, China; ^4^ Research Laboratory for Biomedical Optics and Molecular Imaging, CAS Key Laboratory of Health Informatics, Shenzhen Institutes of Advanced Technology, Chinese Academy of Sciences, Shenzhen, China

**Keywords:** Cu_7.2_S_4_/5MoS_2_, composite nanoflowers, photothermal conversion efficiency, multimodal imaging, synergetic therapy

## Abstract

Transition-metal chalcogenide compounds with facile preparation and multifunctional elements act as ideal photothermal agents for cancer theranostics. This work synthesizes Cu_7.2_S_4_/5MoS_2_ composite nanoflowers and investigates the crystal growth mechanism to optimize the synthesis strategy and obtain excellent photothermal therapy agents. Cu_7.2_S_4_/5MoS_2_ exhibits a high photothermal conversion efficiency of 58.7% and acts as a theranostic nanoplatform and demonstrated an effective photothermal–chemodynamic–photodynamic synergetic therapeutic effect in both *in vitro* and *in vivo* tests. Moreover, Cu_7.2_S_4_/5MoS_2_ shows strong photoacoustic signal amplitudes and computed tomographic contrast enhancement *in vivo*. These results suggest a potential application of Cu_7.2_S_4_/5MoS_2_ composite nanoflowers as photo/H_2_O_2_-responsive therapeutic agents against tumors.

## Introduction

The mortality rate of cancer is expected to increase to 12 million by 2030, which increases the global concern about the effects of therapy (Cleary et al., 2014). Effective cancer treatments to increase patient survival are urgently required. At present, radiotherapy (RT), chemotherapy, and high intensity focused ultrasound (HIFU) treatments (Chen et al., 2019; Khezri et al., 2019; Dubinsky et al., 2020) are the main clinical therapy approaches used to kill tumor cells and prolong the lives of patients. However, the serious side effects and poor prognosis caused during these treatments are significant. Photothermal therapy (PTT), with the advantages of high targeted location and minimal invasiveness, has received much attention in recent years (Jonathan and Yu-Chie, 2013). Plenty of photothermal dominant multifunctional agents, such as noble metal–based nanomaterials (Huang et al., 2006; Howaili et al., 2021), transition metal sulfides (Hu et al., 2019; Huang et al., 2019), carbon-based nanomaterials (Liang et al., 2014; Xuan et al., 2019), and conducting polymers (Zhang W. et al., 2019; Li et al., 2021), have been proposed to markedly promote the therapeutic effect. In addition, the unique physiochemical properties of inorganic nanosystems, including a tunable optical bandgap, controllable composition content, and desirable biocompatibility, provide excellent theranostic performances in oncological applications (Yang et al., 2018).

Medical imaging is a critical technique in achieving a highly effective therapy, particularly in determining the exact location of the tumor prior to treatment (He et al., 2019). Moreover, real-time imaging has been used in clinical diagnostics and targeted therapeutics of tumors. As a result, constructing a well-designed nanoplatform that has therapy and imaging functions is important for nanomedicine in the future. Recently, semiconductor materials, which are low cost, have tunable morphology, and are easily functionalized, have been developed as promising photothermal agent candidates (Chou et al., 2013; Liu et al., 2019; Zhu et al., 2021). Notably, copper sulfide, which has an excellent photothermal conversion efficiency (PCE), good biocompatibility, and catalase-like activities, has attracted attention. In recent years, our research group has made several important achievements in the fabrication and bio-application of CuS superstructures (Tian et al., 2011b), Cu_9_S_5_ plate-like nanocrystals (Tian et al., 2011a), Cu_7_S_4_ (Song et al., 2014), and Cu_7.2_S_4_ nanocrystals (NCs) (Li et al., 2014). Moreover, studies of nanocomposites and multiple compounds of copper sulfide have been carried out (Liu et al., 2015; Huang et al., 2017; Zhang X. et al., 2019; Huang et al., 2019). Meanwhile, in view of the strong absorbance in the near-infrared (NIR) region, molybdenum disulfide (MoS_2_) has raised public concerns in cancer therapy (Liu et al., 2014; Xu et al., 2017; Li et al., 2019). MoS_2_-based composites have been shown to have effective tumor ablation performances and have attracted attention in diagnostics/therapeutics. Typically, MoS_2_ composites have been prepared using a first-hand approach by growing nanoparticles directly on an MoS_2_ nanosheet substrate (Wang et al., 2015b). As with self-doped copper chalcogenides, copper molybdenum sulfides (CMSs) with many copper vacancies are revealed in NIR absorption, which act as p-type semiconductors (Chang et al., 2019). In addition, CMS nanostructures have potential application in computed tomography (CT) imaging owing to the X-ray absorption ability of Mo (Yin et al., 2014; Li et al., 2019). However, most previous reports about CMS nanostructure synthesis were based on chemically exfoliated MoS_2_ nanosheets using a top-down strategy, which is time-consuming and lacks controllable morphology and thicknesses. By aiming for a multifunctional cancer therapy and modality, a direct construct of MoS_2_-based nanocomposite platforms will significantly improve the curative effect of solid tumors.

Herein, we synthesized a PEGylated Cu_7.2_S_4_/5MoS_2_ composite with a flower-like structure *via* a facile one-pot solvothermal method using a bottom-up approach by controlling the proportion of the Cu and Mo precursors. The as-synthesized Cu_7.2_S_4_/5MoS_2_-PEG composite nanoflowers (CSMS-PEG CNFs) were abundant in Cu deficiencies and exhibited an intense optical absorption in the NIR region, which indicates an effective PTT agent and a PT/photoacoustic (PA) imaging contrast agent. The X-ray absorption ability of Mo gives the CSMS-PEG CNFs a computed tomography (CT) imaging ability. At the same time, the catalase-like and glutathione peroxidase–like activities of the Cu_7.2_S_4_ and MoS_2_ result in chemodynamic therapy (CDT) and photodynamic therapy (PDT) functions. Moreover, the integration of the therapy and imaging promotes their potential applications in tumor treatment and, furthermore, nanomedicine.

## Materials and Methods

### Materials

Copper (II) acetate and ammonium molybdate were obtained from Macklin Biochemical Co., Ltd. Thioacetamide and polyethylene glycol 400 (PEG-400) were purchased from Aladdin Industrial Corporation. Deionized water was purified using the Milli-Q system (Millipore Co., United States ). Absolute ethyl alcohol and hydrogen peroxide (30%) were sourced from Sinopharm Chemical Reagent Co., Ltd. Phosphate-buffered saline (PBS) was sourced from Hyclone. All the reagents were used without further purification.

### Synthesis of Cu_7.2_S_4_/5MoS_2_-PEG Composite Nanoflowers and Cu_7.2_S_4_/CuS and MoS_2_ Nanomaterials

In a Teflon-lined autoclave (100 ml), ammonium molybdate (0.58 mmol) and thioacetamide (2.3 mmol) were dispersed in a mixed solvent (30 ml of H_2_O and 30 ml of PEG-400). After stirring for 0.5 h, copper acetate (1.2 mmol) was introduced and stirred for another 0.5 h. Subsequently, the autoclave was kept at 200°C for the corresponding reaction time. After washing several times with water and ethyl alcohol, the samples were collected. Cu_7.2_S_4_/CuS and MoS_2_ nanomaterial was obtained using the same method as Cu_7.2_S_4_/5MoS_2_-PEG composite nanoflowers but without the use of the Mo or Cu precursor, respectively.

### Characterization

The morphology and structural characterization were performed using a scanning electron microscope (SEM, Hitachi S-4800, Japan) and a transmission electron microscope (TEM, JEM-2100F, Japan). X-ray diffraction (XRD) was performed using an Empyrean X-ray diffractometer (PANalytical, Netherlands). Dynamic light scattering (DLS) analysis was carried out using a Malvern Zetasizer (Nano ZS ZEN3700). UV–vis–NIR spectra were recorded from 300 to 1,100 nm using a UV–vis–NIR spectrophotometer (LAMBDA 1050+, PerkinElmer). The concentrations of Mo, Cu, and S were determined by inductively coupled plasma (ICP) atomic emission spectroscopy (Leeman Labs Prodigy). Laser Raman spectra (LRS) were collected using a Horiba spectrometer equipped with a 514-nm incident wavelength laser (LabRAM HR Evolution, Japan). An ESCALab 250Xi (Thermo Scientific) spectrometer was used to measure X-ray photoelectron spectroscopy (XPS). Thermogravimetric analysis (TG) was carried out using a PerkinElmer Pyris Diamond at 10 C min^−1^ in an Ar atmosphere. A Nicolet 6700 spectrophotometer (Thermo Scientific) was used to acquire the Fourier transform infrared (FTIR) spectra. Irradiation treatments were performed using an 808-nm laser (Xilong, Shanghai) with a tunable power density of 0–2 W cm^−2^. Photoacoustic imaging was performed using the customized NIR-II acoustic-resolution photoacoustic microscopy (AR-PAM) system.

### Photothermal Effect

Photothermal conversion performances of the CSMS-PEG CNF dispersions with different concentrations (0–200 μg ml^−1^) were evaluated using an 808-nm laser (1.0 W cm^−2^). Dispersions (600 μl) were irradiated at a distance of 10 cm with a spot diameter of 5 mm. To record the real-time temperature, a thermal imaging camera (FLIR A300, United States ) was used. The recycling experiment was performed to assess the photostability of the CSMS-PEG CNFs by turning the laser on or off for five cycles after cooling down or 5 min of irradiation.

### Photothermal Conversion Efficiency Test

According to our previous reports and Roper’s method, η was calculated using Eq. 1 (Roper, et al., 2007; Guan et al., 2018) as follows:

$$\eta =\frac{hS\left(T_{max}-T_{surr}\right)-Q_{dis}}{I\left(1-10^{-A_{\lambda }}\right)},$$ (1)

where *T*_max_ is the maximum equilibrium temperature under irradiation, *T*_*surr*_ is the room temperature, *A*_λ_ is the absorbance (0.938) at 808 nm, and *I* denotes the laser power density. (*T*_max_ *—* *T*_*surr*_) is 22.6°C, calculated according to Figure 4C. *Q*_*dis*_ denotes the heat loss caused by the absorption of the solvent and the container and was calculated to be 3.2 mW. *S* is the surface area of the container and *h* is the heat transfer coefficient. The value of *hS* was calculated using Eq. 2, in which *C*_*D*_ is 4.2 J g^−1^ C^−1^ (the heat capacity of the solvent) and *m*_*D*_ is 0.6 g (the weight of the dispersion), as shown below:

$$\tau_{s}=\frac{m_{D}C_{D}}{hS}.$$ (2)

Here, τ_*s*_ obtained from Figure 4C refers to the sample system time constant acquired using Eqs 3, 4 as shown below:

$$t=-\tau_{s}ln\theta ,$$ (3)

$$\theta =\frac{T_{surr}-T}{T_{surr}-T_{max}}.$$ (4)

Here, *t* refers to the time starting from the laser-off moment during the whole cooling down process, and θ is a dimensionless parameter scaled using the maximum system temperature.

### *In Vitro* Cytotoxicity Evaluation and Cellular Uptake

4T1 cells were obtained from Hunan University and cultured in high-glucose (4,500 mg L^−1^ glucose) Dulbecco’s modified Eagle medium (DMEM; HyClone) supplemented with 1% penicillin–streptomycin (PS; HyClone) and 10% fetal bovine serum (FBS; HyClone) in a humidified 5% CO_2_ atmosphere at 37°C. The cells were seeded into 96-well plates with 1×10^4^ cells/well for 24 h. Then, they were incubated with the CSMS-PEG CNF dispersions at various concentrations (0, 25, 50, 75, 100, 150, and 200 μg ml^−1^) for another 24 h. Finally, the cell viabilities were evaluated using the cell counting kit-8 (CCK-8; Beyotime) assay according to Eq. 5, as shown below:

$$cell\;viability\;\left(\text{\%}\right)\;=\;\frac{OD\;\left(CSMS-PEG\;NFs\right)}{OD\;\left(none\right)\;}\;\times 100.$$ (5)

Here, *OD (CSMS-PEG CNFs)* means the optical density values obtained using CSMS-PEG CNFs, and *OD (none)* means the optical densities in the blank group. In the cellular uptake experiment, 4T1 cells were seeded into 12-well plates and cultured for 24 h. Then the CSMS-PEG-FITC (fluorescein isothiocyanate) dispersion was added to the cells and incubated for another 12 h. Then, the PBS solution and 4% paraformaldehyde were used to wash and fix the 4T1 cells. An Olympus IX73 microscope (Tokyo, Japan) and a Nikon C2 confocal laser scanning microscope (CLSM) were used to acquire all fluorescent images.

### Extracellular O_2_ Concentration Detection and ^1^O_2_ Detection

For O_2_ production by the CSMS-PEG CNFs, H_2_O_2_ with different concentrations (25, 50, 75, and 100 mM) was added to the CSMS aqueous solution (100 μg ml^−1^). The generated concentration of O_2_ was monitored every 1 min using a portable dissolved oxygen meter (HANNA HI9147). ^1^O_2_ was detected using a singlet oxygen sensor green (SOSG; Invitrogen) reagent in PBS buffer. SOSG solution (2.5 μM) was added to the CSMS-PEG CNF PBS suspension (100 μg ml^−1^, 3 ml). Then the mixture was exposed to an 808-nm laser (1.0 W cm^−2^) for 5 min with and without H_2_O_2_. In addition, after excitation at 488 nm, the emission spectrum between 500 and 600 nm of the mixture was collected using an RF-6000 fluorescence spectrophotometer (Shimadzu, Japan).

### Cellular Reactive Oxygen Species Detection

For the cellular ROS generation detection, 2′,7′-dihydrofluorescein-diacetate (DCFH-DA; Beyotime) fluorescence staining was used. 4T1 cells were seeded into 24-well plates with 5×10^4^ cells/well and incubated in basic culture medium with or without the CSMS-PEG CNF suspension (100 μg ml^−1^), in addition to H_2_O_2_ (100 μM) or L-buthionine sulfoximine (10 μM, L-BSO; Invitrogen). After the cells were cultured for 24 h, fresh DMEM containing 10 μM of DCFH-DA was added in the dark for 20 min. The fluorescence images were acquired using a fluorescence microscope, with excitation at 488 nm and emission between 515 and 540 nm.

### *In Vitro* Live–Dead Cell Experiments

The 96-well plates were coated with 1 × 10^4^ 4T1 cells per well and cultured for 24 h. The cells were then incubated with the CSMS-PEG CNF dispersions at different concentrations (0, 50, 100, and 200 μg ml^−1^) for another 24 h. After irradiation using an 808-nm laser (1.0 W cm^−2^) for 8 min, CCK-8 assay was used to evaluate the vital rates of cells. The cells were also examined under a fluorescence microscope. Calcein acetoxymethyl ester (calcein AM, green fluorescence) and propidium iodide (PI, red fluorescence) co-staining performances were designed to obtain the cell apoptosis observation.

### *In Vivo* Antitumor Efficacy

All animal experiments were conducted in accordance with the “Guide for the Care and Use of Laboratory Animals” from the Institute of Laboratory Animal Resources and were approved by the ethics committee of Shenzhen University. BALB/c nude mice (4–6 weeks old) were purchased from Beijing Vital River Laboratory Animal Technology Co., Ltd. and used under specified pathogen-free (SPF) conditions. 3 × 10^6^ 4T1 murine breast cells (in 30 μl PBS) were injected subcutaneously to establish the tumor models. The mice-born tumors (with a diameter up to approximately 5 mm) were assigned to four groups: i) PBS, ii) PBS + NIR, iii) CSMS-PEG CNFs, and iv) CSMS-PEG CNFs + NIR. PBS solution (50 μL) or the CSMS-PEG CNF PBS suspension (100 μg ml^−1^) was injected intratumorally. After 0.5 h of standing, the experimental mice were exposed to an 808-nm laser (1.0 W cm^−2^) for 10 min. The tumor size was calculated following Eq. 6 as shown below and measured every two days, as was the body weight:

$$tumor\;volume=\left(tumor\;length\right)\times {\left(tumor\;width\right)}^{2}/2.$$ (6)

*V/V*_*0*_ was calculated to assess the cancer therapy effect (*V*_*0*_ denotes the primary tumor volume before irradiation).

### Photothermal and Photoacoustic Imaging

The CSMS-PEG CNF PBS suspension (100 μg ml^−1^, 50 μl) was injected intratumorally into one side of the bilateral tumor–bearing mouse, and the PBS solution (50 μl) was injected into the other side of the tumor for comparison. During 808-nm laser irradiation, the real-time temperature of each mouse was recorded every 2 s using a thermal imaging camera. To investigate the *in vivo* PA performance of the CSMS-PEG CNFs in the subcutaneous tumor, the mouse was placed in the lateral position after being anesthetized *via* an intraperitoneal injection of 10% chloral hydrate. Before and after CSMS-PEG CNF PBS solution injection, the PA signals of the tumor were collected using the customized NIR-II acoustic-resolution photoacoustic microscopy (AR-PAM) system.

### Computed Tomography Imaging

CT imaging of various CSMS-PEG CNF dispersions (0, 1, 2, 4, 8, and 16 mg ml^−1^) was conducted using a SkyScan 1276 CT high-resolution *in vivo* X-ray microtomograph at a voltage of 80 kV (Bruker, Germany). For *in vivo* CT imaging, the CSMS-PEG CNFs (4.0 mg ml^−1^, 100 μL) were injected intratumorally into the tumor-bearing mouse during the period of narcosis. By keeping the mouse untreated for 0.5 h, CT imaging was carried out.

### Statistical Analysis

All data displayed as the arithmetical mean ± standard deviation (SD) were obtained from at least three independent and replicated experiments. Statistical significance was assessed using Origin 2019, for which the significance level was *p* < 0.05. Significant differences are indicated by pentastars in the corresponding figures.

## Results and Discussion

### Preparation and Characterization of Cu_7.2_S_4_/MoS_2_-PEG

**FIGURE 1:**
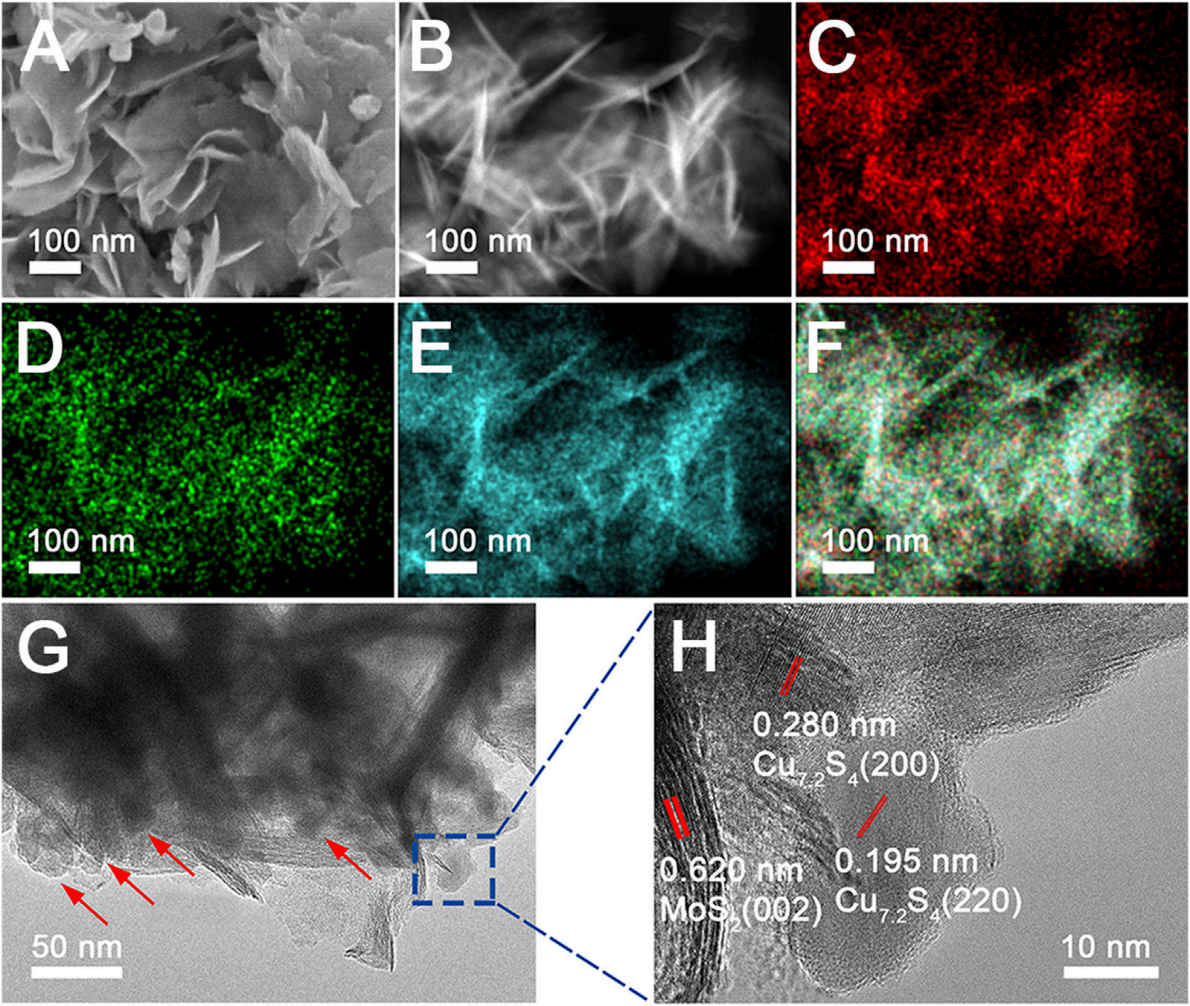
**(A)** SEM image and **(B–F)** HAADF-STEM image and element mapping of the Cu, Mo, and S, as well as overall maps of the CSMS-PEG composite. **(G–H)** TEM and HRTEM images of the CSMS-PEG CNFs.

CSMS-PEG CNFs were synthesized using a one-pot bottom-up approach using a solvothermal method in which transition metal precursors and thioacetamide react in the presence of deionized water and PEG at 200°C for 24 h. As shown in the SEM images (Figure 1A), the obtained CSMS-PEG CNFs had a nanosheet-constructed nanoflower morphology with a length of approximately 200 nm, whose hydrated diameter was approximately 255 nm, obtained using a light scattering technique (Supplementary Figure S1), and whose zeta potential was −24 mV. The high-angle annular dark-field scanning transmission electron microscopy (HAADF-STEM) image in Figure 1B clearly shows the flower-like structure of the CSMS-PEG. Elemental mapping was performed to further investigate the homogeneous distribution of the Cu, Mo, and S elements in the composite, as confirmed by Figures 1C–F. Moreover, typical Cu_7.2_S_4_ nanoparticles with an average diameter of approximately 20 nm decorated the MoS_2_ nanosheet surface (Figure 1G). The size distribution histogram of Cu_7.2_S_4_ nanoparticles is given in Supplementary Figure S2. The lattice fringes and interface of the multilayered MoS_2_ and Cu_7.2_S_4_ could be observed in the high-resolution TEM images (Figure 1H), indicating the crystallinity of the CSMS-PEG CNF heterostructure. The set of lattices with an interlayer spacing of 0.62 nm corresponds to the (002) planes of hexagonal MoS_2_ (Ha et al., 2017), and two sets of lattice fringes with a spacing of 0.280 and 0.195 nm were ascribed to the (200) and (220) planes of Cu_7.2_S_4_, respectively (Li et al., 2014; Lu et al., 2018).

**FIGURE 2:**
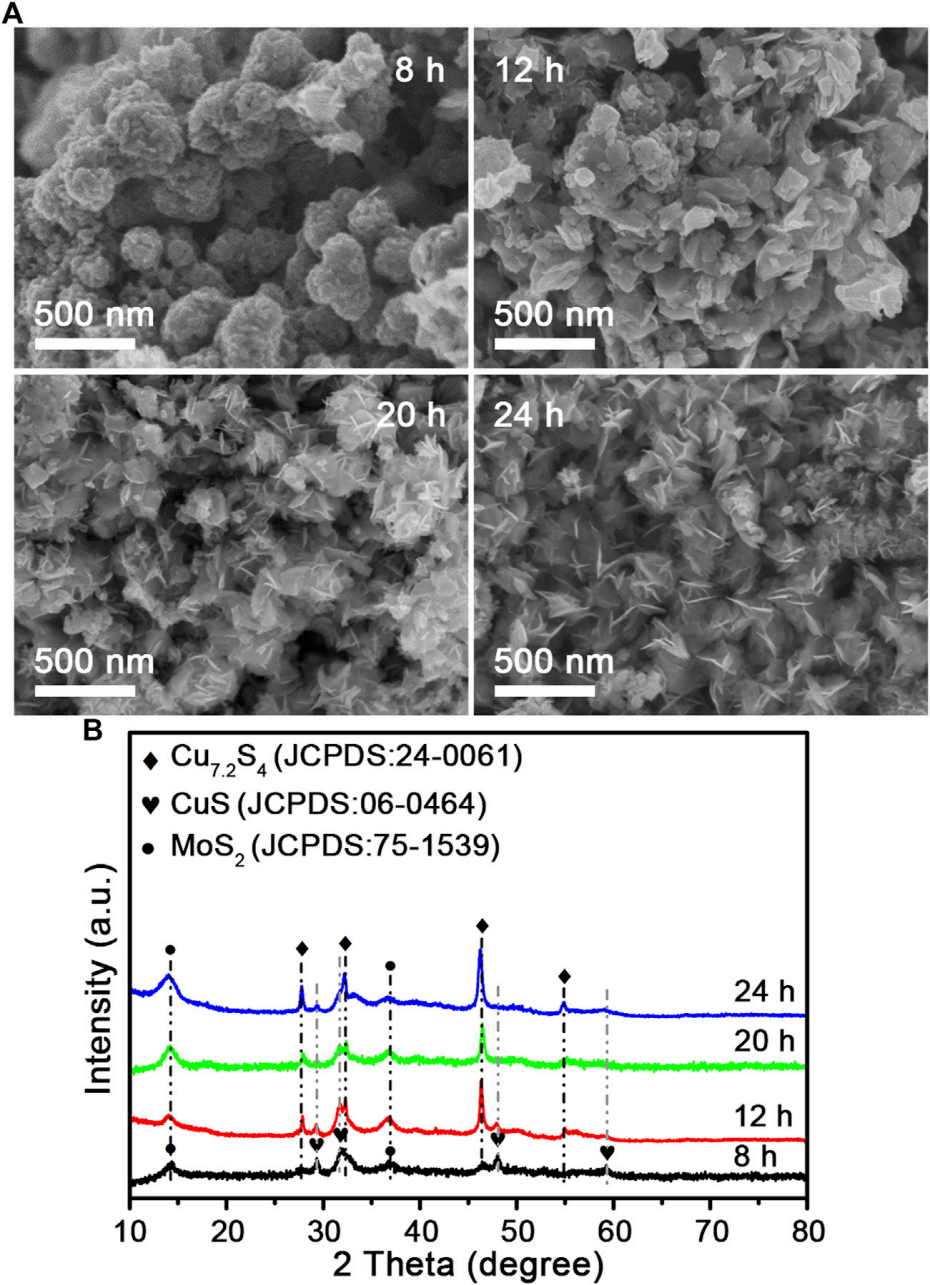
**(A)** SEM images and **(B)** their power XRD pattern of the CSMS-PEG CNFs prepared at 200°C for 8, 12, 20, and 24 h, respectively.

Morphology and structure are key factors that can lead to different optical properties. An effect of the Mo and Cu precursor dosage on composition (Supplementary Figure S3) and morphology (Supplementary Figure S4) was studied, which confirmed the formation process of the Cu vacancies oriented by the MoS_2_ component, and the MoS_2_ nanosheets anchored Cu_7.2_S_4_ nanoparticles. The optimal amounts of ammonium molybdate and copper acetate were 0.58 and 1.2 mmol, respectively. To understand the growth process of the molybdenum disulfide–based composite nanoflowers, we carried out a detailed time-dependent reaction process to observe the CSMS-PEG growth. Figure 2 shows the SEM images and the XRD pattern of molybdenum disulfide composition prepared at different reaction times at 200 C. After the reaction had taken place for 8 h, nanoparticles comprising CuS (JCPDS card no. 06-0464), Cu_7.2_S_4_ (JCPDS card no. 24-0061), and MoS_2_ (JCPDS card no. 75-1539) (Figure 2B) without a regular morphology were observed, as shown in Figure 2A. On extending the reaction time to 12 h, the nanosheet morphology was observed. Consequently, the CuS component was reduced to Cu_7.2_S_4_ with the Cu vacancy, which was confirmed by the appearance of the diffraction peaks occurring at 27.9, 32.3, 46.4, and 55.0° in the relevant XRD patterns. Finally, uniform MoS_2_ nanosheets that anchored Cu_7.2_S_4_ nanoparticles were obtained after 24 h of reaction, according to SEM observation. As expected, the corresponding XRD pattern revealed the coexistence of MoS_2_ and Cu_7.2_S_4_. The PT effects of the CSMS-PEG CNFs prepared with different reaction times (Supplementary Figure S5) were measured under the same conditions. As the 3D flower morphology grew from 8 to 24 h, the photothermal conversion of the CSMS-PEG CNFs improved, which was attributed to the enhanced NIR absorbance of the 3D structures. Thus, 24 h was chosen as an optimal reaction time.

A morphology evolution could be elucidated in three stages: ⅰ) nucleation: ammonium molybdate, copper acetate, and thioacetamide formed a supersaturated solution, and a burst of nucleation resulted in a rapid color change from colorless to dark brown. MoO_4_^2−^ and Cu^2+^ reacted with thioacetamide to form copper and molybdenum sulfide. ⅱ) Growth: according to the different concentrations of polyethylene glycol and the precursor in the solvent, the diameter, thickness, and crystallinity of the nanosheets were oriented by the augmented solvent viscosity and the nanosheet growth rates (Wang et al., 2015a). ⅲ) Delayed Ostwald ripening and aggregation: the high temperature and pressure substantially triggered the Ostwald ripening and enabled the small MoS_2_ nanosheets to shrink and dissolve. This spontaneous self-assembly process was driven by kinetic stability, which led to a substantial reduction of the surface free energy (Zhang et al., 2009; Guan et al., 2018). Finally, the van der Waals interaction between ultrathin MoS_2_ nanosheets composed of few-layered MoS_2_ crystal clusters promoted the 3D nanoflower assembly, which has been reported previously (Fang et al., 2019).

**FIGURE 3:**
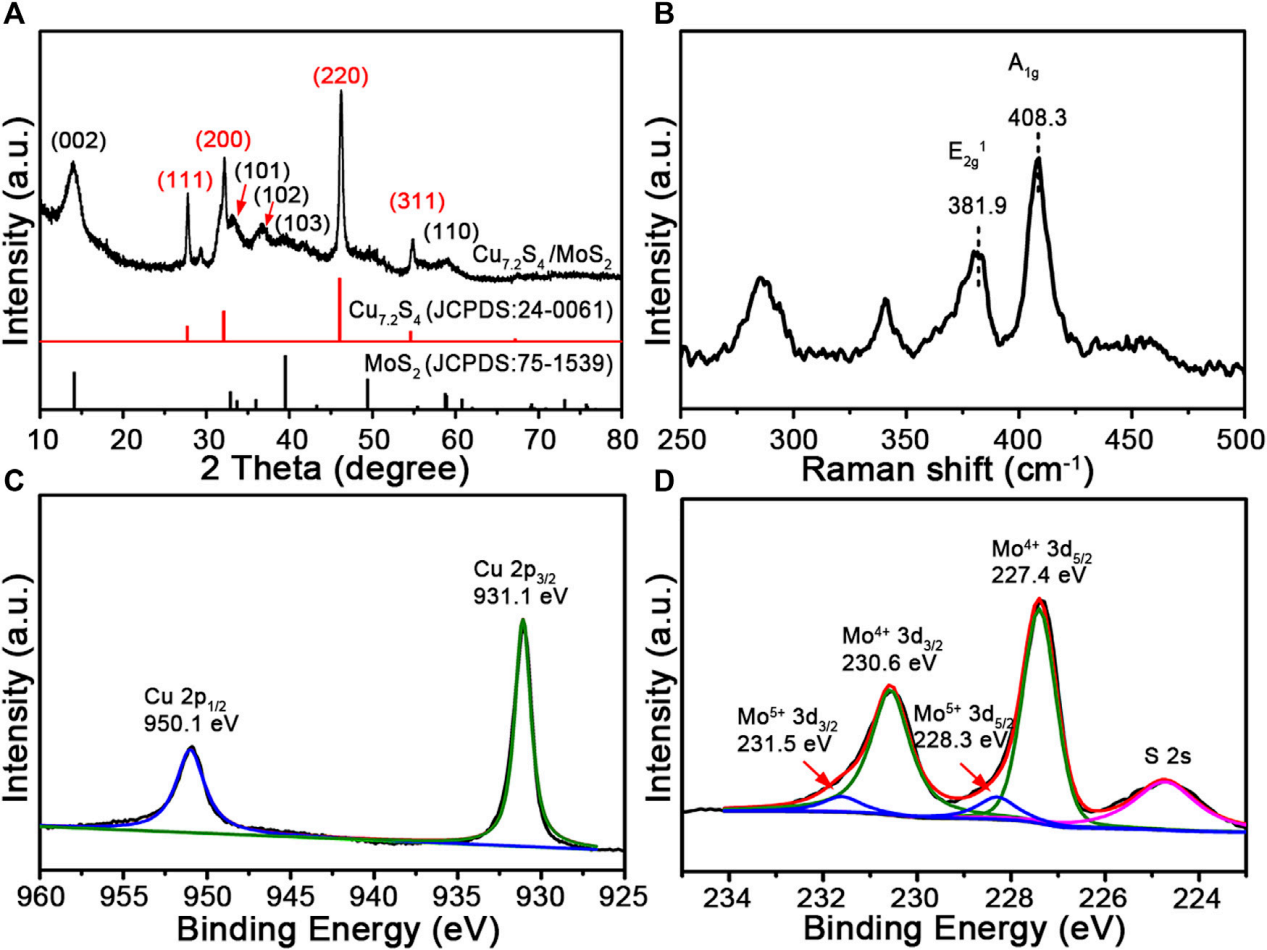
**(A)** XRD pattern, **(B)** Raman spectrum, **(C)** Cu 2p, and **(D)** Mo 3d XPS survey spectra of the CSMS-PEG CNFs.

To further investigate the specific components of the composite nanoflowers, X-ray diffraction (XRD) analysis was performed. As shown in Figure 3A, the representative diffraction peaks match the pattern of MoS_2_ (JCPDS card no. 75-1539) and Cu_7.2_S_4_ (JCPDS card no. 24-0061). Notably, there were no additional peaks in the pattern except for these characteristic peaks. Raman spectroscopy has been widely used in studying transition-metal dichalcogenides. Raman spectra peaks of the CSMS-PEG CNFs (Figure 3B) were assigned to the in-plane E^*1*^_*2g*_ mode at 381.9 cm^−1^ and the out-of-plane A_*1g*_ mode at 408.3 cm^−1^, with Δk = 26.4 cm^−1^, which is in agreement with the multilayered MoS_2_ previously reported (Lee et al., 2010) and consistent with the TEM images. Furthermore, the chemical states of the CSMS-PEG CNF sample were investigated by XPS. As shown in Figure 3C, the fitted peaks located at 931.1 and 950.1 eV could be assigned to the Cu 2p_3/2_ and 2p_1/2_ orbits of the Cu^+^ state (Xu et al., 2016), respectively. The spectrum of Mo 3d (Figure 3D) consisted of Mo 3d_5/2_ at 227.4 eV and Mo 3d_3/2_ at 230.6 eV, which corresponds to Mo^4+^ in MoS_2_ (Lai et al., 2016; Yang et al., 2015). Location peaks at approximately 228.3 and 231.5 eV with a 3.2 eV spin-orbit splitting could be assigned to the relatively small amount of Mo^5+^, which is incompletely sulfurized Mo oxide species (Qin et al., 2014). Notably, owing to the variations of the chemical environment caused by the surface-anchored PEG chains, the binding energy of Cu 2p, Mo 3d, and S 2s showed a slight decrease (Wang et al., 2015b). Furthermore, the peak at 1,060 cm^−1^ in Supplementary Figure S6A was assigned to a typical ether-group stretching vibration of PEG and confirmed the successful PEGylation on the surface. The TG curve of the CSMS-PEG CNFs (Supplementary Figure S6B) revealed a weight loss of 7.0% from 100 to 350°C, which could be attributed to the decomposition of the grafted PEG chains (Wang et al., 2015a). Table 1 exhibits the surface and bulk compositions of the CSMS-PEG CNF sample, which were calculated from XPS data, ICP, and energy dispersive X-ray spectroscopy (EDS) analysis. The atomic ratios of Cu, Mo, and S (29.3, 20.7, and 50.0%) in the bulk were approximately equal to their distribution (28.5, 21.3, and 50.2%) at the surface, which demonstrated the homogeneous component of CSMS-PEG CNFs. In theory, the Cu_7.2_S_4_/5MoS_2_ composition with the corresponding atom percentage of 27.5% Cu, 19.1% Mo, and 53.4% S was fitted perfectly.

**TABLE 1:** Experimental and theoretical compositions of the CSMS-PEG CNFs.

Element	Theoretical	Experimental		
	Cu_7.2_S_4_/5MoS_2_	XPS	ICP	EDS
Cu	27.5	28.5	29.3	36.0
Mo	19.1	21.3	20.7	12.9
S	53.4	50.2	50.0	51.1

### PT Properties of Cu_7.2_S_4_/5MoS_2_-PEG

**FIGURE 4:**
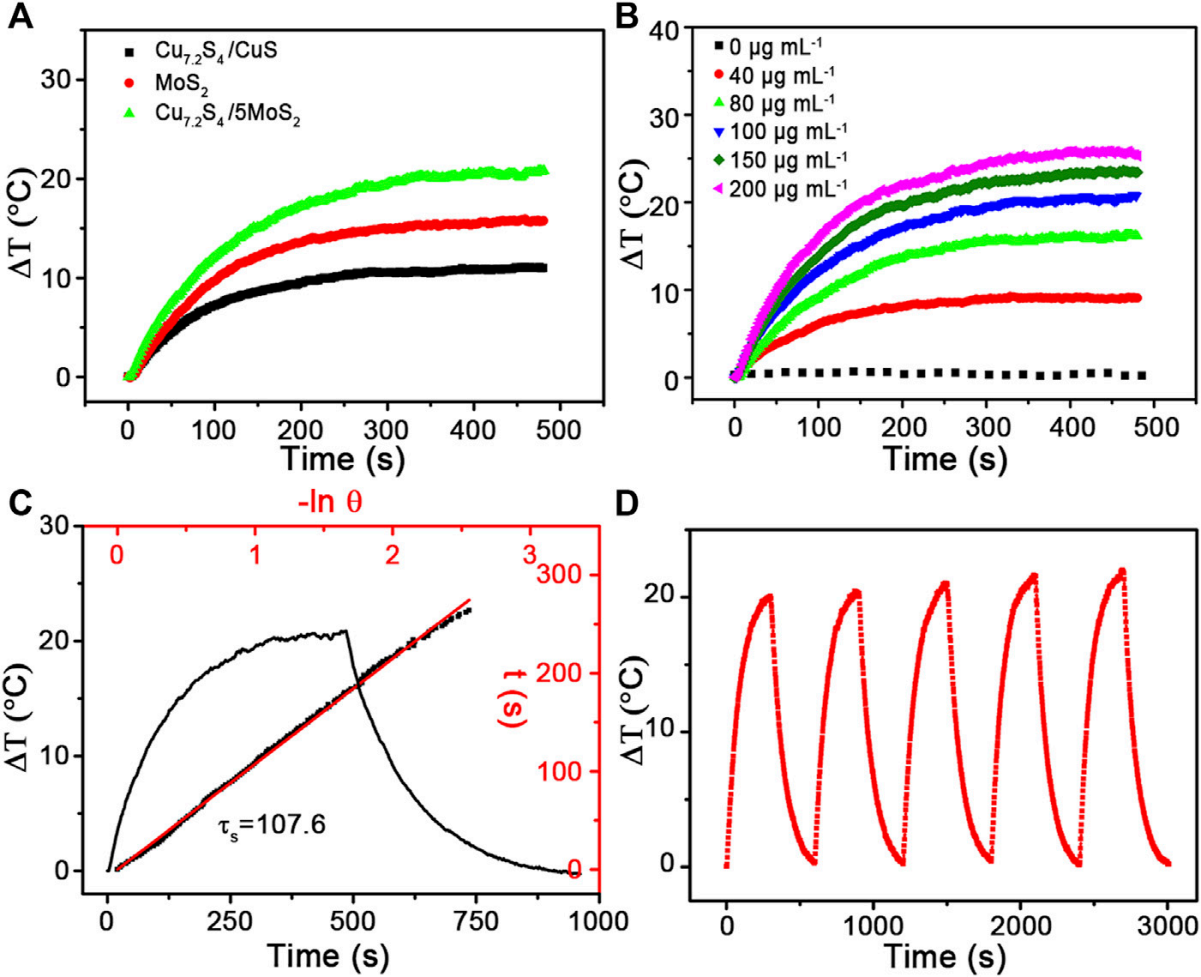
Temperature elevation of the aqueous dispersions of **(A)** Cu_7.2_S_4_/CuS, MoS_2_ and CSMS-PEG CNFs and **(B)** the aqueous dispersions of the CSMS-PEG CNFs with different concentrations (i.e., 0, 20, 40, 60, 80, 100, 150, and 200 μg ml^−1^) under the irradiation of the 808-nm laser (1.0 W cm^−2^) vs. time. **(C)** Real-time temperature curve of the CSMS-PEG CNFs’ suspension with 8 min of heating under an 808-nm laser (1.0 W cm^−2^) and then after cooling down to room temperature, and the time constant for heat transfer (τ_*s*_ = 107.6). **(D)** Photothermal stability of the CSMS-PEG CNFs (100 μg ml^−1^) under irradiation for 300 s (808 nm, 1.0 W cm^−2^) and then with shutting off the laser for five cycles.

The broad and strong NIR absorbance intensity was assigned to copper vacancies arising from the unusual defect structure in our previous study (Li et al., 2015). The vis–NIR absorption spectra of the CSMS-PEG CNF aqueous dispersions with different concentrations were measured (Supplementary Figure S7). The absorbance intensity of the CSMS-PEG CNFs at 808 nm fitted a linear relationship vs. concentration. The PT conversion performances of the pure MoS_2_, Cu_7.2_S_4_/CuS, and the CSMS-PEG CNF (100 μg ml^−1^) aqueous dispersions were measured under laser irradiation (808 nm, 1.0 W cm^−2^) for 8 min. As we can see in Figure 4A, the temperature of the pure MoS_2_ and Cu_7.2_S_4_/CuS suspension increased by 15.8 and 11.1°C, respectively. Significantly, the CSMS compound suspension showed a final temperature increase of up to 20.9°C under the same conditions. The improved photothermal conversation of the CSMS-PEG CNFs might arise from its three-dimensional structure and the abundance of Cu defects, which could contribute to improving the reflectance and absorbance of laser photons (Tian et al., 2011b; Xiao et al., 2016; Cheng et al., 2018).

To demonstrate the relationship of the PT effect and the CSMS-PEG CNFs dosage, aqueous dispersions with various concentrations (0, 40, 80, 100, 150, and 200 μg ml^−1^) were prepared. Under the same laser irradiation of 808 nm, the higher CSMS-PEG CNF concentration resulted in a larger temperature increase (Figure 4B). A concentration of 100 ppm showed a temperature increase of 20.9°C, whereas the temperature of distilled water increased by only 0.7°C under the same conditions. According to this result, the PT conversion efficiency was calculated as 58.7% (Figure 4C) (Guan et al., 2018; Cao et al., 2019). Furthermore, the laser on-off circulation test results are displayed in Figure 4D. The maximum temperatures and Δ*T* remain unchanged during the five cycles, indicating the high stability of the CSMS-PEG CNFs under photo-irradiation. In addition, the CSMS-PEG CNFs showed a good dispersibility in water, saline, PBS, and DMEM without observation of aggregation (Supplementary Figure S8). These results demonstrate that the CSMS-PEG CNFs are a potential PTT agent with a high stability and photothermal conversion efficiency.

### *In Vitro* O_2_ and ROS Generation of Cu_7.2_S_4_/5MoS_2_-PEG

Aside from their roles as a PT agent, copper sulfides and molybdenum sulfides can also stimulate ROS generation through the Fenton-like and catalase-like reaction shown in the following processes (Chang et al., 2019; Badrigilan et al., 2020):

$${\text{Cu}}^{+}/{\text{Mo}}^{4+}+{\text{H}}_{2}{\text{O}}_{2}\to {\text{Cu}}^{2+}/{\text{Mo}}^{6+}+\text{OH}+{\text{OH}}^{-},$$

$${\text{Cu}}^{2+}/{\text{Mo}}^{6+}{+2\text{H}}_{2}{\text{O}}_{2}\to {\text{Cu}}^{+}/{\text{Mo}}^{4+}+{\text{O}}_{2}+{2\text{H}}_{2}\text{O}\text{.}$$

**FIGURE 5:**
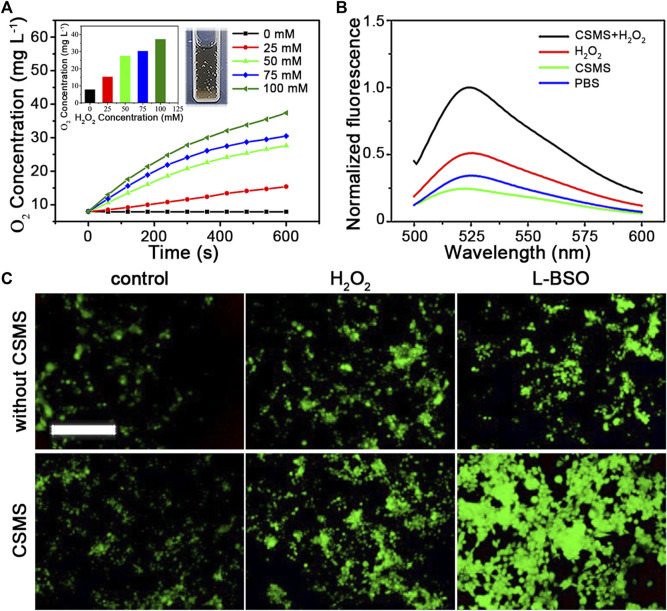
**(A)** O_2_ generation in the CSMS-PEG CNFs’ dispersion (100 μg ml^−1^) with different H_2_O_2_ concentrations vs. time. Insert: final dissolved oxygen concentration and picture of oxygen production in the CSMS-PEG CNFs’ suspension in the presence of H_2_O_2_. **(B)** Normalized fluorescence emission spectrum of SOSG (2.5 μM) incubated in the CSMS-PEG CNFs’ PBS suspension (100 μg ml^−1^) with or without H_2_O_2_ after 808-nm laser irradiation (1.0 W cm^−2^) (Ex = 488 nm). **(C)** Fluorescence images of 4T1 stained with DCFH-DA (10 μM) with different incubation treatments (scale bar = 500 μm).

Herein, we quantified the generation of O_2_ triggered by the CSMS-PEG CNFs on exposure to H_2_O_2_ using a dissolved oxygen meter. The CSMS-PEG CNF suspension (100 μg ml^−1^) maintained a low dissolved oxygen level (7.9 mg L^−1^) in the absence of H_2_O_2_ (Figure 5A). After the addition of H_2_O_2_ (25 mM) to the CSMS-PEG CNF PBS suspension, the dissolved oxygen concentration increased from 7.9 to 15.4 mg L^−1^ within 10 min. Moreover, the oxygen yield was proportionable to the H_2_O_2_ amount, which confirms that CSMS-PEG CNFs are an effective agent for O_2_ generation. This could overcome the hypoxia microenvironment inside a tumor and improve cancer therapy efficacy (Yin et al., 2019).

To evaluate the generation of ^1^O_2_ induced by the CSMS-PEG CNFs, SOSG was added. As shown in Figure 5B, the PBS solution containing only SOSG exhibited weak fluorescence. Owing to an intensive absorption of the CSMS-PEG CNF suspension at 525 nm (Supplementary Figure S7A), the fluorescence intensity of the SOSG at 525 nm slightly decreased after irradiation treatment in the CSMS-PEG CNF dispersion. Furthermore, we proposed a test that focused on the dependence between ROS generation and H_2_O_2_ concentrations. Compared with the control groups with only CSMS-PEG CNFs or added H_2_O_2_, the fluorescence intensity of the SOSG in the CSMS-PEG CNF suspension with H_2_O_2_ at 525 nm increased to 409.8% under the same conditions.

To directly observe a chemodynamic effect, intracellular ROS generation was stained with DCFH-DA that could be rapidly oxidized by ROS due to the fluorescent 2′,7′-dichlorofluorescein (DCF) acting as a probe for oxidative stress (Chang et al., 2019). As shown in Figure 5C, the DCF fluorescence in 4T1 cells exposed to CSMS-PEG CNFs was much higher than that in cells cultured in normal DMEM, which indicates that CSMS-PEG CNFs could generate more ROS in cancer cells. H_2_O_2_ (100 μM) was added to the cells to simulate the tumor microenvironment, and the fluorescence intensity of DCF with CSMS-PEG CNF incubation was higher than that without CSMS-PEG CNFs. In addition, the *in vitro* cytotoxicity of H_2_O_2_ at different concentrations (0, 25, 50, 75, 100, 150, and 200 μg ml^−1^) was tested, confirming a high cell viability with the addition of H_2_O_2_ at concentrations less than 100 μg ml^−1^ (Supplementary Figure S9). Referring to a report on a glutathione peroxidase–like activity of high-valence Mo, we proposed that Mo^5+^ is reduced by glutathione (GSH) in the following process (Chang et al., 2019):

$${\text{Mo}}^{5+}+\text{GSH}\to {\text{Mo}}^{4+}+\text{GSSG}\text{.}$$

L-BSO, an inhibitor of GSH, was adopted to evaluate the effect of GSH depletion on ROS generation. Figure 5C shows the brighter fluorescence of cells co-incubated with CSMS-PEG CNFs and a nontoxic dose of L-BSO (5 μM) compared with CSMS-PEG CNFs alone. The results revealed that GSH depletion could increase the chemodynamic cytotoxicity of CSMS-PEG CNFs and sensitize tumor cells to a CDT agent. As a result, Cu^+^, Mo^4+^, and Mo^5+^ ion–rich CSMS-PEG CNFs can serve as an effective CDT and PDT agent.

### *In Vitro* Cytotoxicity of Cu_7.2_S_4_/5MoS_2_-PEG

**FIGURE 6:**
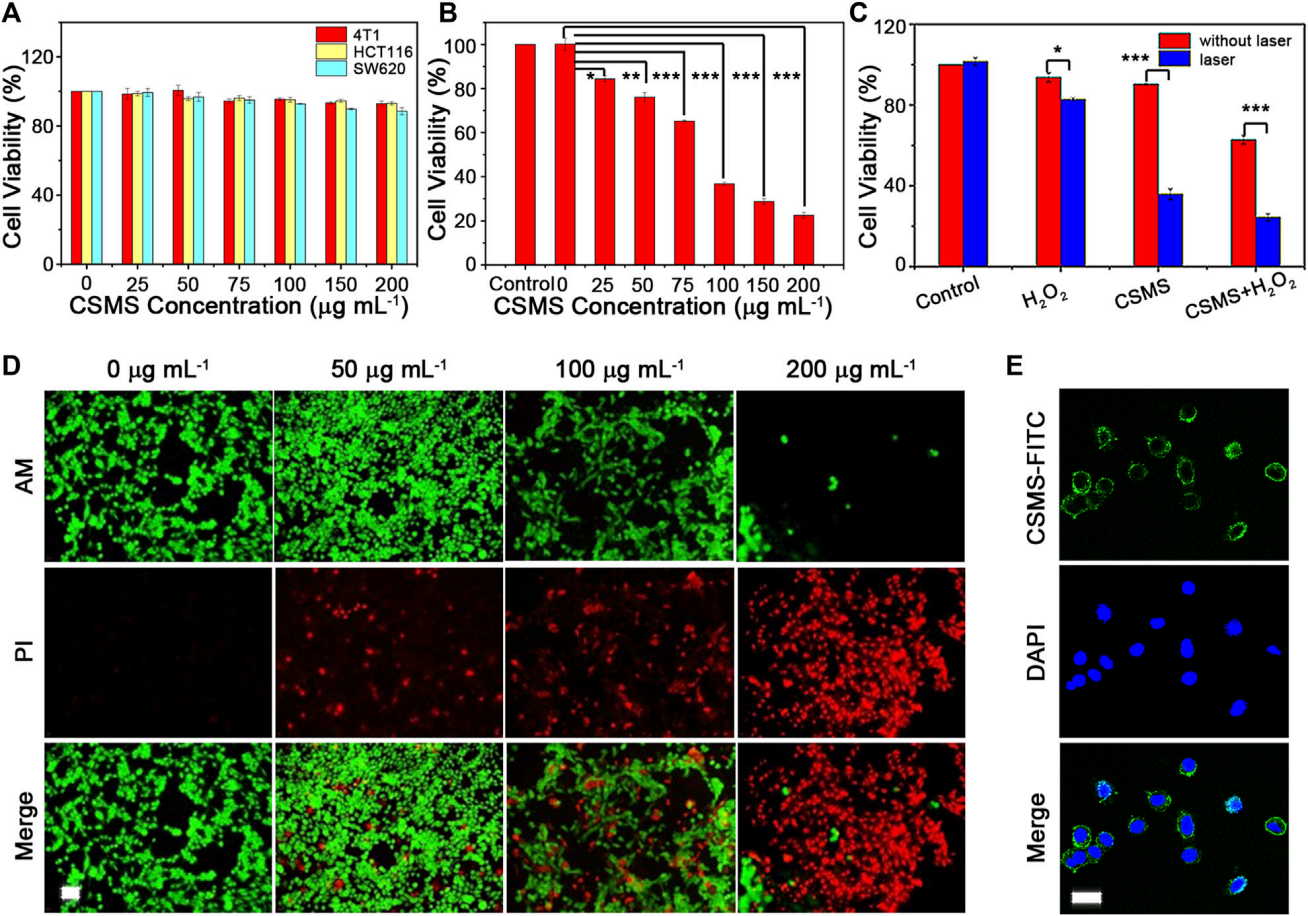
**(A)** Cytotoxicity of the CSMS-PEG CNFs under 4T1, HCT116, and SW620 cell strain incubation. **(B)** Photothermal injury of tumor cells in various concentrations of the CSMS-PEG CNFs upon 808-nm laser irradiation (1.0 W cm^−2^, 8 min). **(C)** Relative viabilities of tumor cells after incubation with the CSMS-PEG CNFs (100 μg ml^−1^) with or without the addition of H_2_O_2_ and 808-nm laser irradiation (1.0 W cm^−2^, 8 min). **(D)** Fluorescence images of calcein AM/PI–stained 4T1 cells upon 808-nm laser irradiation (1.0 W cm^−2^, 8 min) (calcein AM = 2.0 μM; PI = 10 μg ml^−1^; alive, green; dead, red; scale bar = 50 μm). **(E)** Fluorescence images of cellular uptake (DAPI, blue; FITC, green; scale bar = 20 μm) (^★^ *p* < 0.05, ^★★^ *p* < 0.01, ^★★★^ *p* < 0.001).

4T1, HCT116, and SW620 cells were incubated with a CSMS-PEG CNF solution for 24 h at different concentrations (i.e., 0, 25, 50, 75, 100, 150, and 200 μg ml^−1^) and then tested using CCK-8 assay. As shown in Figure 6A, the cell viability of all the three cell types remained over 88%, demonstrating the low cytotoxicity and excellent biocompatibility of the CSMS-PEG CNFs *in vitro*. The photothermal cytotoxicity of the CSMS-PEG CNFs was evaluated using irradiation from an 808-nm laser (1.0 W cm^−2^) for 8 min (Figure 6B). With increasing concentration, the cells could be efficiently killed in the presence of the CSMS-PEG CNFs and laser irradiation. The cell viability was 43% after incubation in the CSMS-PEG CNF suspension (100 μg ml^−1^), whereas 22% of cells lived in the CSMS-PEG CNF suspension (200 μg ml^−1^) upon irradiation. Thus, CSMS-PEG CNFs are an effective photothermal agent. Moreover, the laser-treated 4T1 cells were also co-stained with calcein-AM and PI and then observed by fluorescence microscopy (Figure 6D). To further verify the *in vitro* coefficient of PT–CD–PD treatment of CSMS-PEG CNFs, the 4T1 cells were treated with and without H_2_O_2_ (100 μM) with the laser turned on and off. Compared with the untreated group, the CSMS-PEG CNF and the CSMS-PEG CNFs + H_2_O_2_ groups effectively promote cell death under laser irradiation with cell viability decreasing from 90 to 36% and from 62 to 24%, respectively. As shown in Figure 6C, a rising death rate was observed in the presence of H_2_O_2_, the laser, or both, which verifies the photothermal, chemodynamic, and photodynamic synergistic effect on tumor cell death. Considering the biocompatibility of the CSMS-PEG CNFs, the cellular uptake of the CSMS-PEG CNFs was analyzed with FITC modification and DAPI staining by CLSM (Figure 6E). The DAPI exhibited bright blue fluorescence in the nucleus, whereas the green fluorescence of the FITC in the cytoplasm around the nuclei was observed after incubation with the CSMS-PEG-FITC, which indicates that the nanoparticles were effectively internalized.

### *In Vivo* Tumor Therapy Efficacy of Cu_7.2_S_4_/5MoS_2_-PEG

**FIGURE 7:**
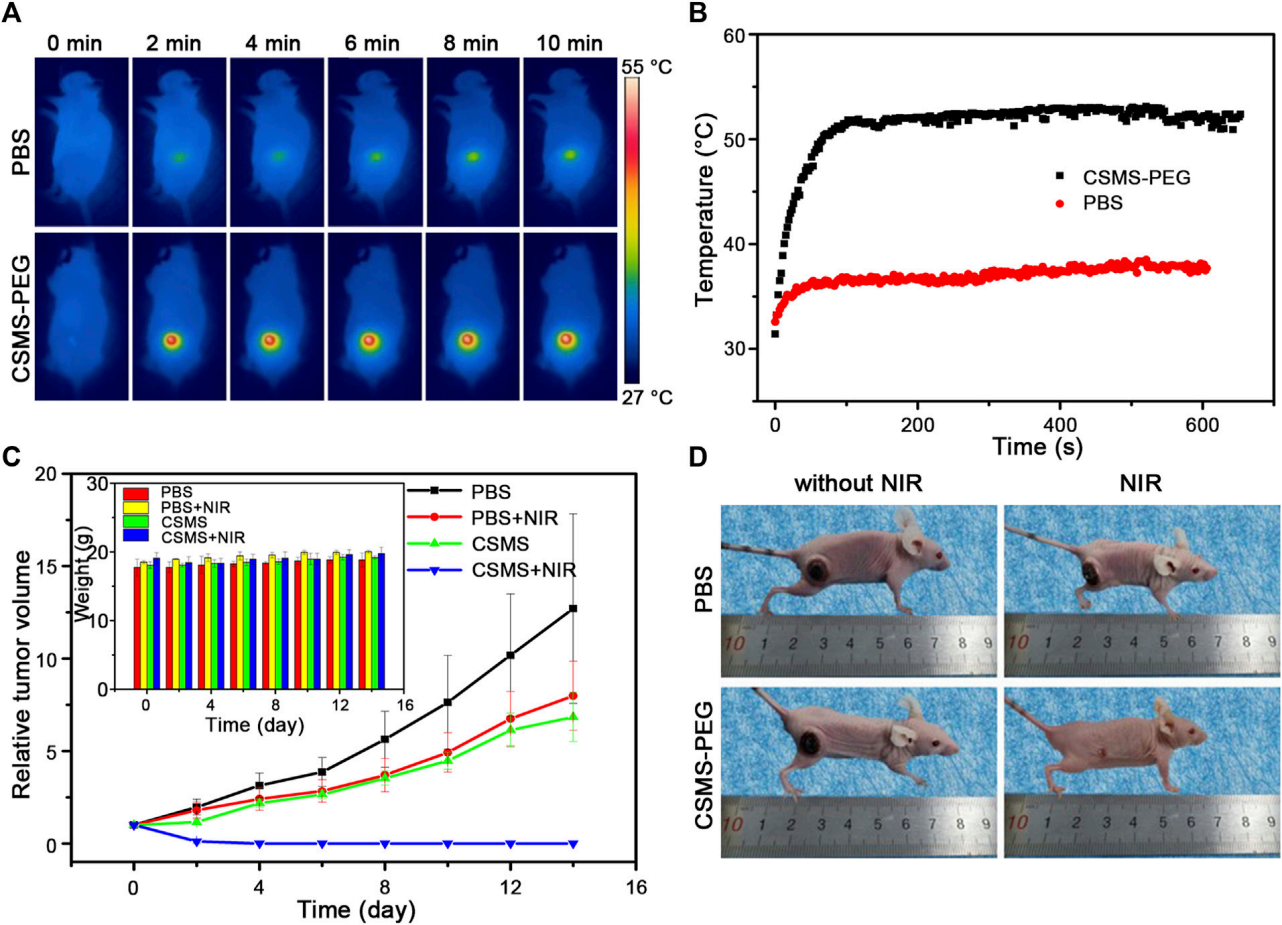
**(A)** Thermal images of tumor-bearing mice under 808-nm laser irradiation (1.0 W cm^−2^) treated with the PBS or the CSMS-PEG CNF suspension injection at different time intervals. **(B)** Real-time temperature change curves of **(A)** vs. time. **(C)** Growth states of tumors and body weight (insert) during treatment. **(D)** Photos of mice taken on the 14th day.

Based on the low toxicity and good photothermal conversion *in vitro*, we assessed the NIR-triggered photothermal behavior *in vivo*. Four groups were designed to evaluate the photothermal therapy efficacy with three mice per group: i) PBS (50 μl) injection alone, ii) PBS (50 μl) injection and 808-nm laser treatment, iii) CSMS-PEG CNF (50 μl, 100 μg ml^−1^) injection alone, and iv) CSMS-PEG CNF (50 μl, 100 μg ml^−1^) injection and 808-nm laser treatment. All treatments were kept uniform with an intratumoral injection and 808-nm laser irradiation (a spot diameter of 10 mm at a power density of 1.0 W cm^−2^ for 10 min). The real-time temperature change was recorded using an infrared camera. As shown in Figure 7A, the temperature of the tumor tissues with the CSMS-PEG CNF injection increased rapidly from approximately 31°C to approximately 49°C within 1 min under laser irradiation, followed by a stable temperature of 52 ± 0.5°C for 8 min. The surface temperature of the tumors vs. time is shown in Figure 7B. In comparison with group (ii), which exhibited a temperature increase of less than 6.0°C and a secure maximum temperature of approximately 38.0°C, the CSMS-PEG CNFs were confirmed to be an efficient photothermal ablation agent of cancer cells. These results indicate that CSMS-PEG CNFs still had a noticeable *in vivo* photothermal performance.

To test whether the health of the mice was affected by the treatments, the body weight and the tumor size were recorded every 2 days. As shown in Figure 7C and the insert, the tumor volume of mice in group (i) and group (ii) grew rapidly and became approximately 12 times and eight times the initial size. Notably, the tumor growth was slightly decreased in the CSMS-PEG CNF dispersion injection without laser irradiation group, which confirms the effectiveness of the chemodynamic treatment in restraining tumor growth to some extent. Moreover, under the coexistence of the CSMS-PEG CNFs injection and laser irradiation, the tumors in group (iv) were gradually eliminated by the 14th day after treatment. The tumor area temperature was approximately 52°C, and the original tumor area presented black scars by the next day. The scars were almost eliminated after 14 days (Figure 7D), which was attributed to the combination of photothermal therapy with chemotherapy, both of which were simultaneously activated using an 808-nm laser. No reoccurrence was observed within 14 days, further demonstrating an excellent therapeutic effect by the CSMS-PEG CNFs. Besides, no obvious change was found in the body weight of mice after these treatments, which indicates the reasonable harmlessness of the CSMS-PEG CNFs and intensity of 808-nm laser irradiation. Herein, a highly effective and feasible therapeutic strategy combined with the CSMS-PEG CNFs and laser irradiation was designed and confirmed.

### Photothermal, Photoacoustic, and Computed Tomography Triple-Modality Images of Cu_7.2_S_4_/5MoS_2_-PEG

**FIGURE 8:**
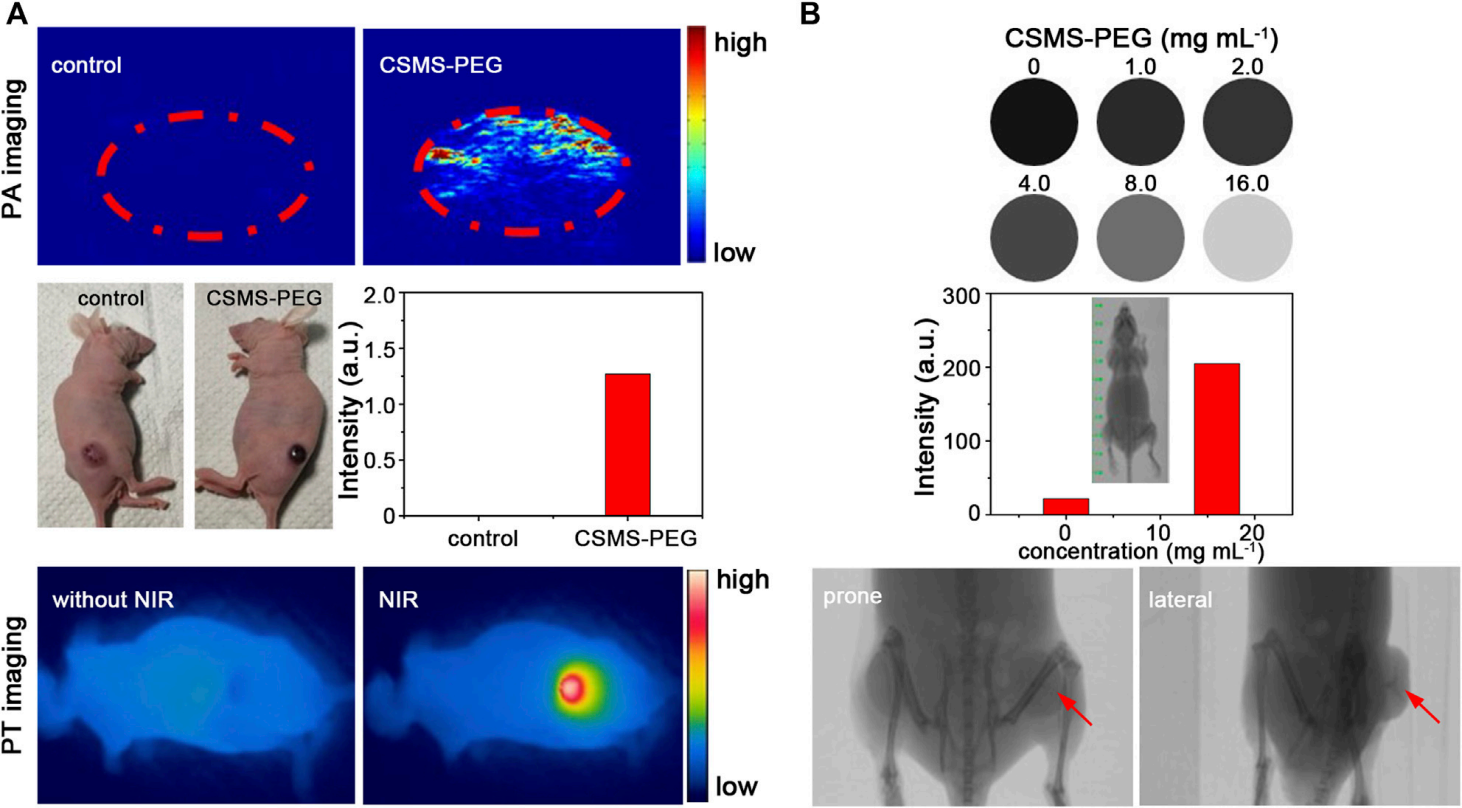
**(A)** PA and PT images of a tumor-bearing mouse with or without the CSMS-PEG CNF injection under 808-nm irradiation, respectively. **(B)** CT signals in response to the CSMS-PEG CNFs with different concentrations and computed tomography scanning images of a bilateral tumor–bearing mouse with the CSMS-PEG CNF injection.

Because of the pressure jump and the ultrasound waves induced by heat, the CSMS-PEG CNFs with an effective photothermal conversion were considered to be a contrast agent for PA imaging. The *in vivo* PA imaging experiments were performed using a pulse laser (808 nm) with a laser fluence rate of 15 mJ cm^−2^, and the PA signals of the tumors were monitored. Compared with the control group, the PA signals were significantly enhanced from approximately 0.0014 to approximately 1.27 a.u. after the CSMS-PEG CNF dispersion injection (Figure 8A). More importantly, by utilizing the large attenuation of the X-rays by Mo, we believed that the CSMS-PEG CNFs can be utilized as an excellent CT imaging contrast agent (Yang et al., 2019). Figure 8B presents the CT images of the CSMS-PEG CNF PBS dispersions with different concentrations. As expected, CT signal intensities were enhanced with increasing concentration. The CT signal intensity of the CSMS-PEG CNFs (16 mg L^−1^) increased by up to 9.3 times compared with that of the CSMS-PEG CNF (1.0 mg L^−1^) aqueous suspension, which corresponds to the exhibition of a stronger contrast in the intratumoral injection tumor than in other soft tissues in the prone and lateral position images (signed with red arrows). These results demonstrated that the CSMS-PEG CNFs possessed an excellent capability as a multifunctional agent of PT, PA, and CT tri-model imaging.

## Conclusion

In summary, the as-synthesized CSMS-PEG CNFs showed good biocompatibility and worked as an effective photothermal agent with a high photothermal conversion performance of 58.7%. Meanwhile, the catalase-like and the glutathione peroxidase–like activities of the Cu_7.2_S_4_ and MoS_2_ components in the composite were unitized as a chemodynamic therapy and photodynamic therapy platform. Moreover, the obvious X-ray absorption ability of Mo gave them a PT/PA/CT imaging function, which could guide the treatment processes. The CSMS-PEG CNFs integrated the PTT–CDT–PDT treatment and the PT/PA/CT imaging as a whole and revealed an effective tumor therapy efficacy. This work constructed a theranostic system for multimodal imaging and PTT-dominant anticancer treatment, which showed a potential for biomedical applications.

## Data Availability

The original contributions presented in the study are included in the article/Supplementary Material; further inquiries can be directed to the corresponding author.
