# Supplementary material for: A Near-Infrared Light Triggered Composite Nanoplatform for Synergetic Therapy and Multimodal Tumor Imaging
Source: Front Chem. 2021 Jul 22;9:695511. doi: 10.3389/fchem.2021.695511 (PMC8339317; doi:10.3389/fchem.2021.695511)
Supplement: Supplementary file 1 [file Presentation1.pdf]

Supplementary material for:

A Near-Infrared Light Triggered Composite Nanoplatform for Synergetic Therapy and Multimodal Tumor Imaging

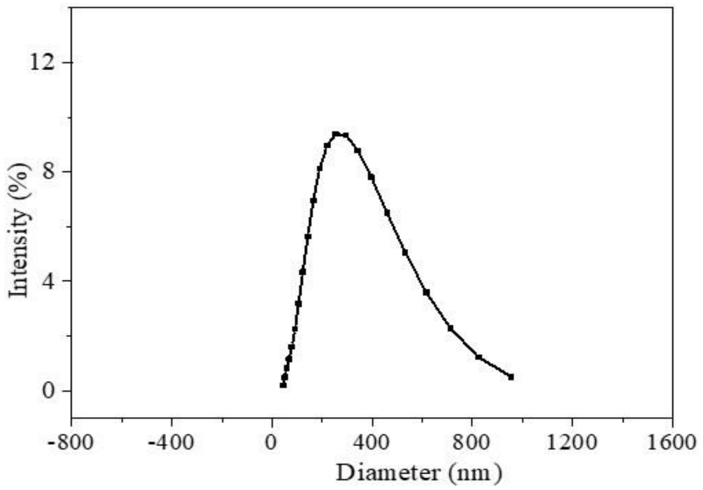

Figure 1. Particle size distribution of the CSMS-PEG suspension in PBS.

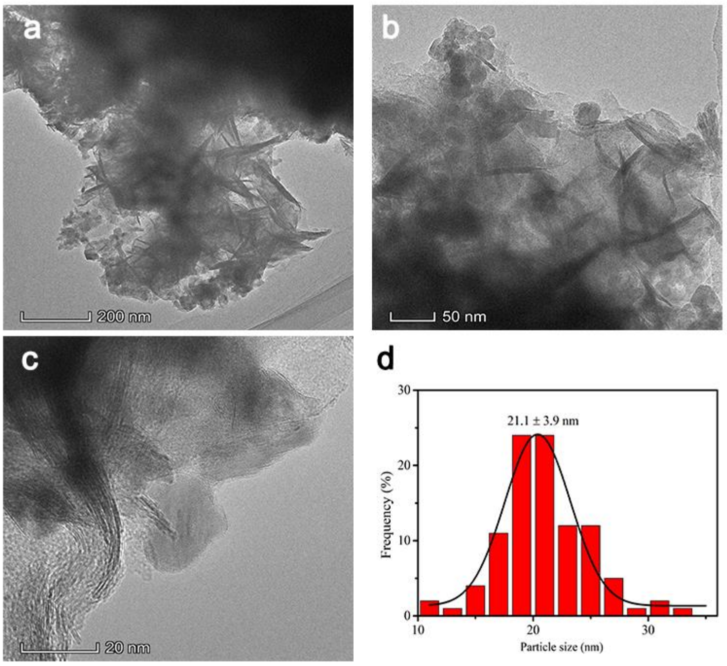

Figure 2. (a-c) TEM images of CSMS nanoflowers. (d) Size distribution histogram of $Cu_{7.2}S_4$ nanoparticles according to TEM images.

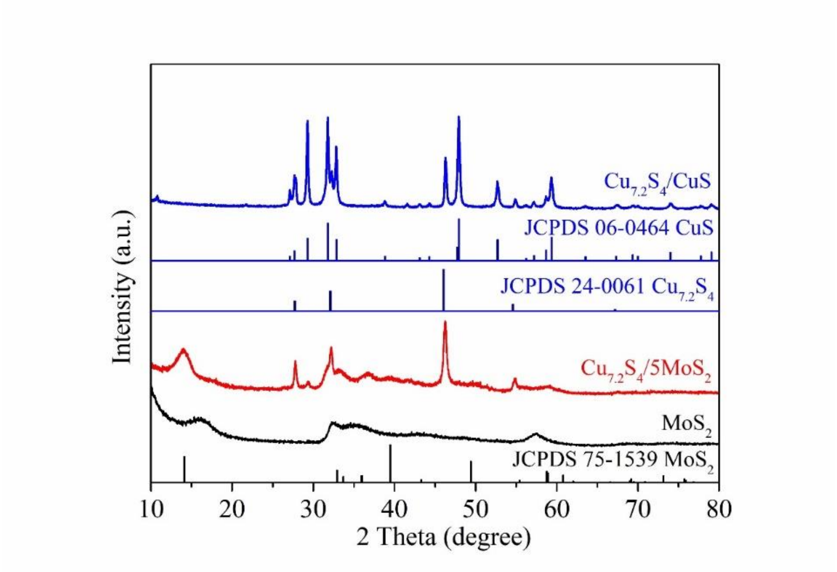

Figure 3. XRD pattern of the MoS_2 (0.58Mo0Cu), $Cu_{7.2}S_4/5MoS_2$ (0.58Mo1.2Cu) and $Cu_{7.2}S_4/CuS$ (0Mo1.2Cu).

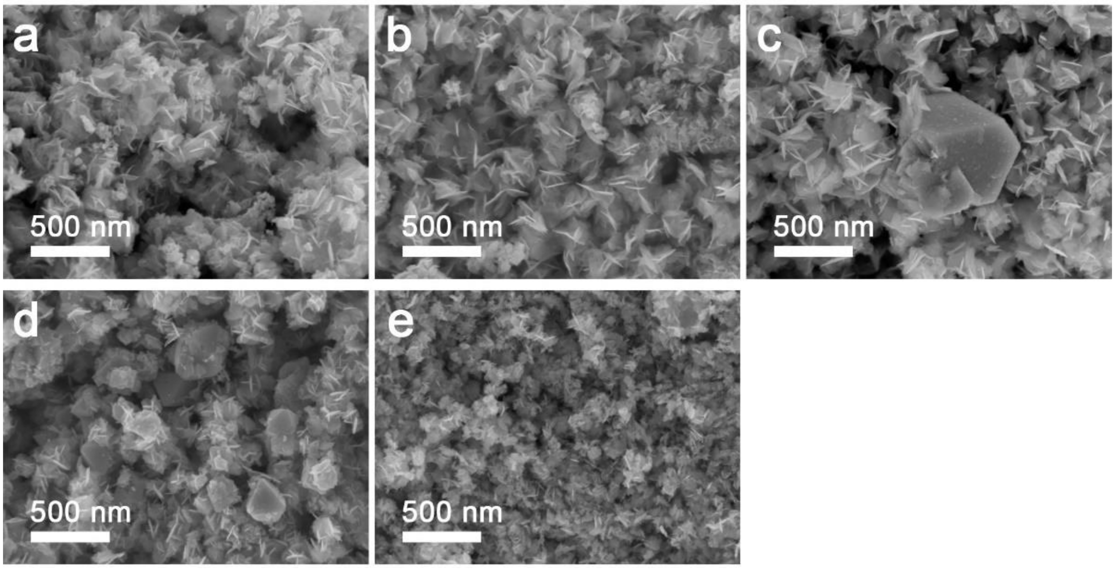

Figure 4. SEM images of the CSMS-PEG prepared with (a) 0.58Mo1.6Cu; (b) 0.58Mo1.2Cu; (c) 0.58Mo0.8Cu; (d) 0.44Mo1.2Cu and (e) 0.29Mo1.2Cu.

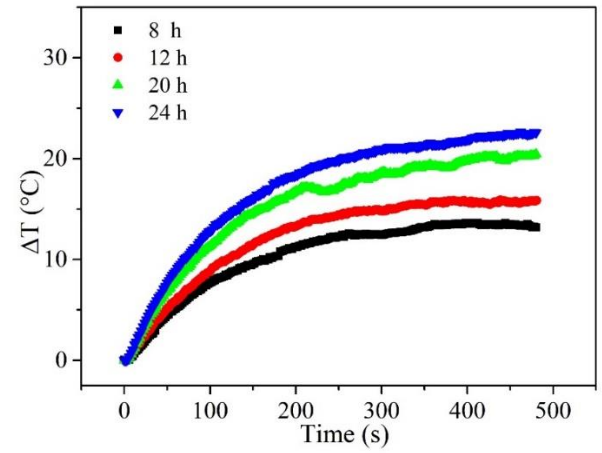

Figure 5. Temperature elevation of the CSMS-PEG CNFs prepared in 200 ℃ for 8, 12, 20 and 24 h under the irradiation of 808 nm laser (1.0 W cm^{-2}) versus time, respectively.

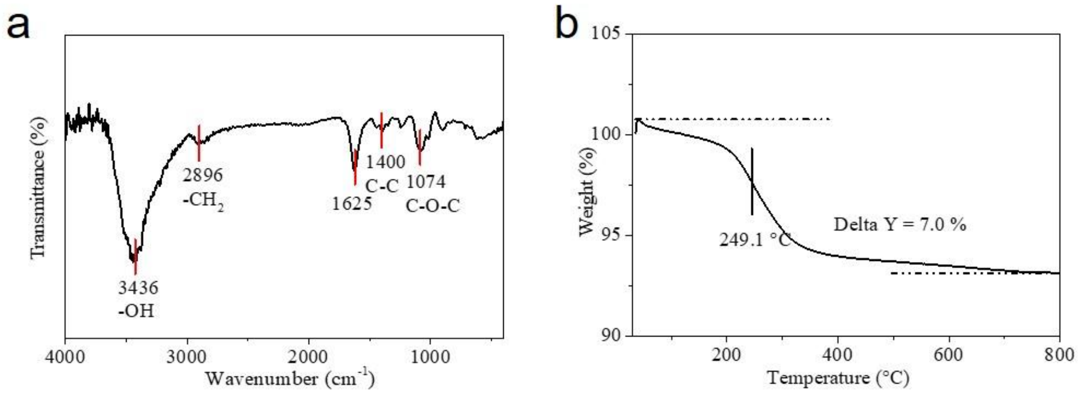

Figure 6. (a) FT-IR spectrum and (b) TG curve of the CSMS-PEG.

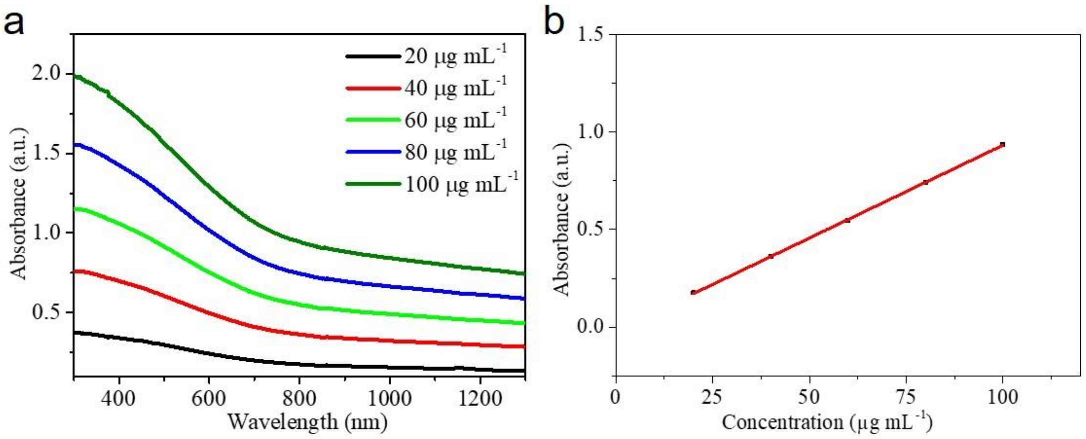

Figure 7. (a) UV-vis-NIR diffraction spectra of CSMS-PEG with 20, 40, 60, 80, and 100μg mL^{-1},

respectively; (b) Absorbance value at 808 nm versus the aqueous dispersion concentration of CSMS-PEG (R^2=0.9997).

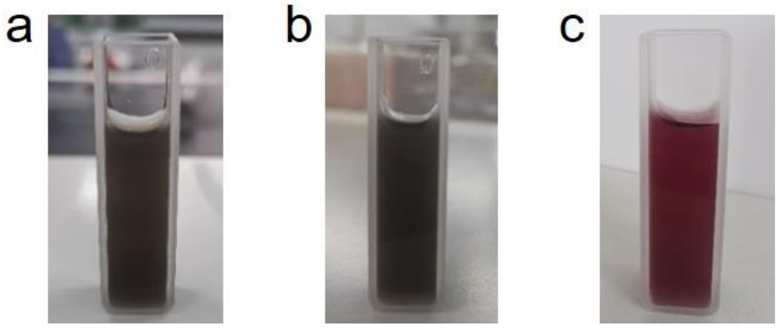

Figure 8. Photos of CSMS-PEG suspension in (a) saline; (b) PBS and (c) DMEM after standing for 48 hours.

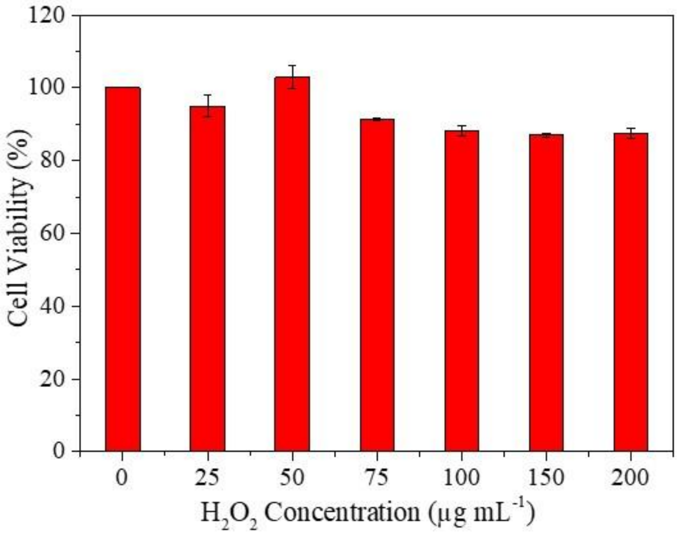

Figure 9. Relative viabilities of tumor cells after incubation with different H_2O_2 concentration (0, 25, 50, 75, 100, 150 and 200 μg mL^{-1}).
